# Baseline Lymphopenia Predicts Survival in ICI-Naïve Solid Tumor Patients Receiving Immune Checkpoint Inhibitors: A Propensity-Matched Real-World Pan-Cancer Analysis

**DOI:** 10.3390/cancers18121940

**Published:** 2026-06-14

**Authors:** Ahmed Ismail, Nina Balanchivadze, George R. Simon, Yanis Boumber

**Affiliations:** 1 Department of Medicine, Eastern Virginia Medical School, Old Dominion University, Norfolk, VA 23507, USA; 2 Virginia Oncology Associates, Norfolk, VA 23502, USA; nina.balanchivadze@usoncology.com; 3 Department of Medical Oncology, Ohio Health, Columbus, OH 43228, USA; george.simon@ohiohealth.com; 4 Department of Medical Oncology, Ohio Health, Delaware, OH 43015, USA

**Keywords:** Immune checkpoint inhibitors, lymphopenia, absolute lymphocyte count, overall survival, healthcare utilization, immune-related adverse events, real-world data, solid tumors, prognostic biomarker

## Abstract

**Background:** Baseline lymphopenia is common among advanced solid tumors and may influence the efficacy/safety of immune checkpoint inhibitors (ICIs), but large real-world evidence is limited. We evaluated the association between baseline absolute lymphocyte count (ALC) and clinical outcomes in adults with solid tumors treated with ICIs in routine practice. **Methods:** We performed a retrospective cohort study using TriNetX. Adults with solid tumors who received pembrolizumab, nivolumab, or atezolizumab (ICI-Naïve) between January 2015 and June 2026 were included. Baseline ALC was measured within 30 days before first treatment and was classified as lymphopenic (ALC < 1.5 × 10^9^/L) or non-lymphopenic (ALC ≥ 1.5 × 10^9^/L). Propensity score matching (1:1) yielded 5249 patients per group. The index date was the first immunotherapy date, and outcomes were assessed at 6, 12, 24, 36 months, and 5 years. The primary outcome was 24-month overall survival (OS); secondary outcomes were OS at 6 and 12 months and 6-month risks of healthcare utilization, immune-related adverse events (irAEs), and serious infections; and exploratory outcomes included OS at 36 months and 5 years. All outcomes were analyzed using Kaplan–Meier analysis, Cox proportional hazards models, and risk ratios. Subgroup analysis included OS stratified by solid tumor subtypes and prior lines of therapy. **Results:** After matching, patients with baseline lymphopenia had consistently worse OS. Compared with patients without lymphopenia, the lymphopenia cohort had lower OS at 6 months (HR 1.29, 95% CI 1.22–1.37), 12 months (HR 1.28, 95% CI 1.21–1.35), 24 months (HR 1.26, 95% CI 1.2–1.33), and, in exploratory analyses with substantial right censoring and limited observed follow-up, 36 months (HR 1.26, 95% CI 1.2–1.33) and 5 years (HR 1.26, 95% CI 1.2–1.33), though these estimates should be considered hypothesis-generating only. At 6 months, baseline lymphopenia was associated with a greater healthcare utilization (RR 1.05, 95% CI 1.02–1.09), a higher infection risk (RR 1.08, 95% CI 1.01–1.15), and similar rates of clinically coded irAEs (RR 1.0, 95% CI 0.93–1.09), an observation subject to competing risk from early mortality in the lymphopenic cohort. Subgroup analysis, stratified by tumor subtypes and prior lines of therapy, showed consistently lower OS in the lymphopenia group, consistent with the primary outcome results. **Conclusions:** In this large propensity-matched real-world analysis of 10,498 patients with diverse solid tumors, baseline lymphopenia at ICI initiation was associated with persistently inferior OS at 6, 12, and 24 months (primary and secondary endpoints), greater early healthcare utilization, and a higher serious infection risk. Critically, lymphopenic patients developed irAEs at an identical rate to non-lymphopenic patients despite worse survival, a dissociation suggesting that baseline ALC stratifies patients along mortality risk and immune activation capacity as partially independent axes. These findings could support the use of baseline ALC as a simple, universally available biomarker that informs not only survival prognosis but also the anticipated toxicity profile of ICI therapy and highlight the need for competing-risk analyses and prospective immune phenotyping to characterize this relationship fully.

## Simple Summary

Many people with advanced cancer have low lymphocyte counts, but it is unclear how this common blood test should influence decisions about immune checkpoint inhibitor treatment. In this study, we used a large United States electronic health record network to examine adults with multiple solid tumor types who received pembrolizumab, nivolumab, or atezolizumab. We compared patients who started immunotherapy with low lymphocyte counts to similar patients with normal counts, carefully balancing other factors that might affect outcomes. We found that patients with low counts before treatment had consistently shorter survival, needed more hospital care early after treatment, and may have had more serious infections, without a clear increase in immune-related side effects. These results suggest that a simple, widely available blood test can help identify higher-risk patients starting immunotherapy and may guide how closely they are monitored and supported.

## 1. Introduction

Immune checkpoint inhibitors (ICIs) targeting programmed cell death-1 (PD-1) and programmed death-ligand 1 (PD-L1) have transformed the management of multiple advanced solid tumors, producing durable responses and survival benefits across various solid malignancies [1,2]. Despite these advances, outcomes with ICIs remain heterogeneous, and there is a continued need for simple, reproducible biomarkers that can stratify prognosis and inform treatment and monitoring strategies in routine practice [3,4,5,6].

Lymphocytes play a central role in antitumor immunity, and both tumor-infiltrating and circulating lymphocyte levels have been linked to cancer prognosis [7,8]. Treatment-related lymphopenia after chemoradiation is common and has been consistently associated with inferior survival across a range of solid tumors, underscoring the importance of systemic immune competence for long-term outcomes [9,10]. Beyond treatment-induced lymphopenia, observational data in general populations indicate that low peripheral lymphocyte counts are associated with increased all-cause mortality, suggesting that baseline lymphopenia may be a marker of impaired immune reserve and vulnerability to adverse outcomes [11,12].

In the context of ICIs, several single-center or tumor-specific studies have evaluated absolute lymphocyte count (ALC) as a potential biomarker. Across solid tumors, pretreatment lymphopenia is observed in roughly one-quarter of patients [13] and has repeatedly been associated with poorer outcomes, including in the immunotherapy era [14]. Still, findings have been mixed and often limited by modest sample sizes and a restricted range of tumor types. For example, a retrospective series of patients receiving ICIs found that baseline lymphopenia did not consistently predict survival [13]. In contrast, dynamic changes in ALC after treatment initiation were more strongly associated with outcomes. Other work has focused on related hematologic indices, such as the neutrophil-to-lymphocyte ratio, which has repeatedly been associated with poorer survival in both general cancer and ICI-treated cohorts [3,4,15]. Still, these composite measures are less intuitive than a single lymphocyte threshold for bedside risk stratification. Critically, much of the current framework in ICI biomarker research implicitly treats immune activation as a shared continuum, in which greater baseline immune competence is associated with both enhanced antitumor efficacy and increased risk of immune-related toxicity, whereas impaired immune competence may predispose to treatment resistance and reduce susceptibility to immune-related adverse events. Whether baseline lymphopenia, a readily available marker of diminished circulating immune reserve, conforms to this paradigm has not been rigorously evaluated at scale.

Large, multi-center, real-world analyses examining baseline ALC across diverse solid tumors treated with ICIs are scarce [16,17,18]. Existing studies are frequently restricted to single tumor types (such as NSCLC or melanoma), specific agents, or highly selected academic populations, limiting generalizability to broader ICI-treated populations encountered in routine practice [19,20]. Furthermore, few studies have simultaneously assessed long-term survival together with healthcare utilization, immune-related adverse events (irAEs), and serious infections, which are critical to understanding the full clinical impact of baseline lymphopenia in this setting [20,21,22].

To address these gaps, we conducted a large retrospective cohort study using the US Collaborative Network within the TriNetX multi-center electronic health record platform, including adults with various solid tumors who were treated with pembrolizumab, nivolumab, or atezolizumab in routine clinical practice. We evaluated the association between baseline ALC, categorized as lymphopenic versus non-lymphopenic, and overall survival at multiple time points, as well as early healthcare utilization, irAEs, and serious infections, to determine whether baseline lymphopenia serves as a simple, clinically actionable prognostic marker in ICI-treated patients with solid tumors.

## 2. Materials and Methods

### 2.1. Data Source and Study Design

We conducted a retrospective cohort study using the US Collaborative Network within the TriNetX federated electronic health record (EHR) platform, which aggregates de-identified clinical data from 67 US health care organizations, including academic centers and community hospitals. The network captures demographics, diagnoses, procedures, medications, and laboratory values in near-real time, enabling large-scale real-world comparative effectiveness research. The study was performed in accordance with the TriNetX governance framework; all data are de-identified, and participating institutions have determined that analyses using this platform do not constitute human subjects research and therefore do not require additional institutional review board approval. Our study was exempt from the institutional IRB.

### 2.2. Study Population

We identified adults (age ≥ 18 years) with a diagnosis of a solid malignancy who received at least one dose of pembrolizumab, nivolumab, or atezolizumab between 1 January 2015 and 7 June 2026, within the US Collaborative Network. Solid tumors were defined using ICD-10 codes for malignant neoplasms of non-hematologic primary sites, and patients with primary hematologic malignancies were excluded (Supplementary Table S1). Approximately 70% of patients in both pre-matched cohorts had ICD-10 codes in the C76–C80 range, which encompasses malignant neoplasms of ill-defined, secondary, and unspecified sites. This distribution reflects the real-world composition of ICI-treated patients across a large multi-site network: individuals with advanced, frequently metastatic disease managed in both academic and community settings, in whom metastatic burden is the dominant clinical reality and for whom primary site documentation may be incomplete. This population is systematically underrepresented in registrational ICI trials, which are enriched for performance-status-selected, biomarker-profiled patients; the prognostic signal of baseline ALC demonstrated here is therefore particularly relevant to patients most commonly encountered in routine practice, for whom a complete blood count may be the most consistently available risk-stratification tool. However, it is important to note that tumor-site classification and ICD-10 codes, including C76–C80, are mutually exclusive.

### 2.3. Index Event and Follow-Up

The index date was defined as the date of first ICI administration during the study period. Baseline covariates were ascertained from all available records before the index date. Patients were followed from the index date until death, the last recorded healthcare encounter, or the end of the relevant analysis window (6, 12, or 24 months), whichever occurred first. The exploratory analysis window included 36 months and 5 years.

### 2.4. Exposure

Baseline absolute lymphocyte count (ALC) was defined as the lymphocyte count measured within 30 days before the index ICI dose. Patients were categorized into two groups based on this value: lymphopenic (ALC < 1.5 × 10^9^/L) and non-lymphopenic (ALC ≥ 1.5 × 10^9^/L). The 1.5 × 10^9^/L threshold was chosen a priori, consistent with prior oncology studies using similar cut-points to define lymphopenia and approximate the lower limit of the normal reference range for circulating lymphocytes. As an exploratory analysis, baseline ALC was additionally defined using a narrower measurement window, lymphocyte count measured within 7 days before the index ICI dose, to assess the robustness of findings to the exposure definition.

### 2.5. Baseline Covariates

Covariates were selected a priori based on clinical relevance and availability in the TriNetX platform. Demographic variables included age at index, sex, race, and ethnicity. Cancer-related variables included primary tumor site (e.g., lung, gastrointestinal, genitourinary, breast, melanoma, other), presence of metastatic disease, and prior exposure to systemic anticancer therapy and radiation before ICI initiation. Comorbidities were captured using ICD-10 codes for major conditions, including diabetes, chronic kidney disease, chronic obstructive pulmonary disease, heart failure, cerebrovascular disease, and cardiovascular disease. Baseline medication covariates included use of systemic corticosteroids, immunosuppressants, antineoplastic agents, antibiotics, and proton pump inhibitors at any time before the index date. Baseline laboratory values (e.g., leukocytes, creatinine, lactate dehydrogenase, and neutrophil count) were also extracted when available to characterize the cohorts; however, they were not included in the propensity score to avoid overfitting and collider bias.

### 2.6. Propensity Score Matching

To reduce confounding by indication, we used 1:1 propensity score matching (PSM) to create comparable cohorts of patients with and without baseline lymphopenia. Separate PSM procedures were performed for each follow-up window (6, 12, 24 months, and the exploratory 36 months, and 5 years) using the TriNetX-built-in nearest-neighbor matching algorithm without replacement, with a caliper of 0.1 on the logit of the propensity score. The propensity score model included age, sex, race, ethnicity, primary tumor site, metastatic status, key comorbidities, prior systemic therapy, prior radiation, and baseline corticosteroid and immunosuppressant use. Balance between cohorts after matching was assessed using standardized differences, with values < 0.1 indicating adequate balance. All outcome analyses were conducted in the matched populations.

### 2.7. Outcomes

The primary outcome was overall survival (OS) at 24 months after ICI initiation, defined as time from index date to death from any cause, with patients censored at last known follow-up within the network. Secondary OS endpoints included OS at 6 and 12 months after ICI initiation, evaluated using time-to-event methods over the corresponding follow-up windows. For each OS outcome, patients with the same outcome (death) before the start of the analysis window were excluded to ensure that only new events were captured. Secondary non-mortality outcomes were assessed over the first 6 months after ICI initiation. Healthcare utilization was defined as any of the following: outpatient visit, home health visit, non-acute visit, inpatient hospitalization, emergency department visit, or ICU admission during the 6-month window, based on procedure and visit codes specified in the TriNetX outcome definitions. irAEs were defined using a prespecified composite of autoimmune and inflammatory diagnoses commonly attributed to ICIs (including colitis, pneumonitis, hepatitis, endocrinopathies, nephritis, and dermatologic reactions), and infections were defined as a composite of pneumonia, sepsis, opportunistic infections, and other severe infectious diagnoses. Exploratory outcomes included OS at 36 months and 5 years after ICI initiation. Additionally, an exploratory comparison of OS between patients receiving PD-1 inhibitors and those receiving PD-L1 inhibitors was performed within the lymphopenic cohort. Prespecified subgroup analyses for OS were conducted by tumor type and by prior lines of therapy. For non-mortality outcomes, irAEs were further examined by individual irAE category to explore potential subtype-specific associations with baseline lymphopenia status.

### 2.8. Statistical Analysis

For the mortality and survival outcomes, time-to-event outcomes (OS at each time point) were analyzed using Kaplan–Meier (KM) survival curves and compared between lymphopenic and non-lymphopenic cohorts using log-rank tests. Hazard ratios (HRs) and 95% confidence intervals (CIs) for death were estimated using Cox proportional hazards models implemented in TriNetX, with lymphopenia status as the main exposure. Proportional hazards assumptions were evaluated using the platform’s Schoenfeld residual-based tests. For binary 6-month non-mortality outcomes (healthcare utilization, irAEs, and serious infections), we calculated risks, risk differences, and risk ratios (RRs) with 95% CIs comparing the lymphopenia and non-lymphopenia cohorts. All analyses were two-sided with a significance threshold of *p* < 0.05; however, given the large sample size and multiple endpoints, emphasis was placed on the magnitude and precision of effect estimates rather than sole reliance on *p*-values. Time-to-event analyses were also performed using KM curves and log-rank tests, with HRs and 95% CIs estimated using Cox proportional hazards models. Specifically for the irAE outcomes, we acknowledge that neither conventional risk ratio analysis nor Cox regression fully accounts for the competing risk of early mortality. The observed estimates should be interpreted with the understanding that higher early mortality in the lymphopenic group reduces ascertainment time for irAEs and that formal competing-risk modeling would be required to fully characterize the subdistribution hazards for this endpoint. All analyses were performed using the TriNetX Analytics platform.

## 3. Results

### 3.1. Patient Selection and Baseline Characteristics

**Figure 1:**
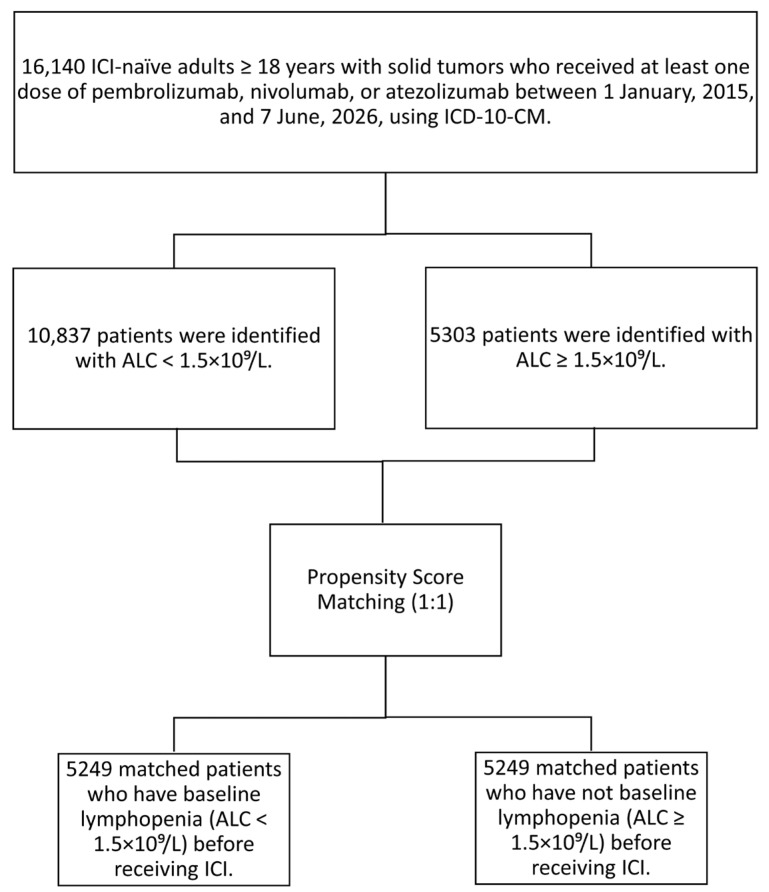
Flowchart of cohort selection and propensity score matching.

A total of 16,140 ICI-naïve adults ≥ 18 years old with solid tumors who received at least one dose of pembrolizumab, nivolumab, or atezolizumab between 1 January 2015 and 7 June 2026 were identified in the US Collaborative Network. After applying cohort definitions and requiring a baseline ALC within 30 days before ICI initiation, 10,837 patients with baseline lymphopenia (ALC < 1.5 × 10^9^/L) and 5303 patients without baseline lymphopenia (ALC ≥ 1.5 × 10^9^/L) were included in the unmatched cohorts. Before propensity score matching, patients with baseline lymphopenia were more likely to have prior radiation (20.9% vs. 11.9%), prior antineoplastic therapy (50.9% vs. 42.7%), and a higher burden of comorbidities, consistent with a more treatment-exposed population; in addition, standardized differences exceeded 0.1 for radiation history, antineoplastic exposure, corticosteroid use, and head and neck tumor site before matching. Baseline laboratory profiles also differed, with the lymphopenia cohort exhibiting higher levels of systemic illness markers. Propensity score matching achieved well-balanced cohorts. For all the follow-up windows, matching yielded 5249 patients per group (10,498 total), with standardized differences < 0.1 for all included covariates, indicating adequate balance across demographics, tumor types, metastatic status, comorbidities, prior therapies, and baseline corticosteroid and immunosuppressant use (Table 1, Figure 1). The visual propensity score distribution before and after matching is shown in Supplementary Figure S1.

### 3.2. Follow-Up Durations

Across all analysis windows, follow-up was relatively short and broadly similar between cohorts, with consistently slightly shorter mean follow-up in the lymphopenia group, reflecting higher early mortality. Median follow-up was approximately 105 days in the lymphopenia cohort and 123 days in the non-lymphopenia cohort and remained stable across all analysis windows, including the exploratory 36-month and 5-year windows, reflecting the high early event rate and the censoring structure of the TriNetX platform, in which follow-up is computed to the last recorded encounter. Long-term survival estimates at 36 months and 5 years therefore derive from Kaplan–Meier extrapolation with substantial right censoring and fewer patients with confirmed long-term observation and should be interpreted as hypothesis-generating rather than confirmatory. Detailed follow-up statistics are provided in Table 2.

### 3.3. Primary Outcome

#### Overall Survival at 24 Months

**Figure 2:**
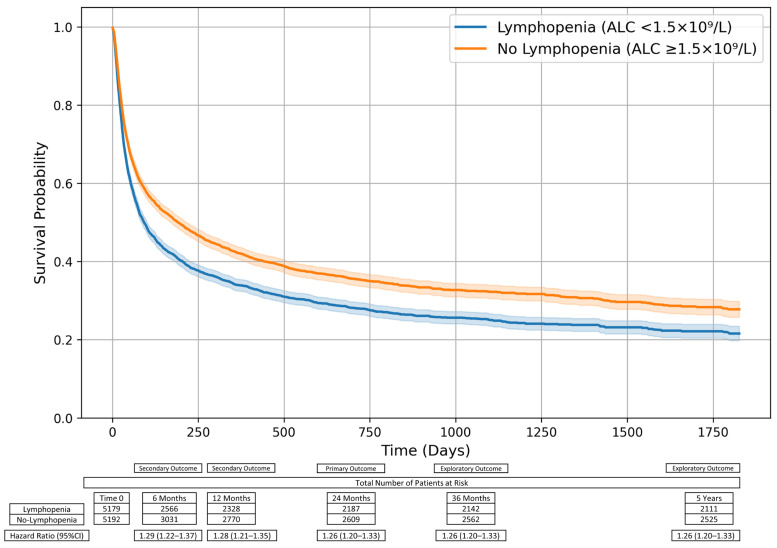
Kaplan–Meier Analysis of Overall Survival in Propensity-Score-Matched Adults with Solid Tumors Treated with Immune Checkpoint Inhibitors, Stratified by Baseline Lymphopenia Status. Kaplan–Meier curves depict overall survival from the date of first immune checkpoint inhibitor (ICI) administration in propensity-score-matched adults with baseline lymphopenia (absolute lymphocyte count [ALC] < 1.5 × 10^9^/L; blue) and without baseline lymphopenia (ALC ≥ 1.5 × 10^9^/L; orange). Shaded areas represent 95% confidence intervals. The number-at-risk table below the *x*-axis shows the number of patients remaining under observation in each cohort at 0, 180, 365, 730, 1095, and 1825 days. The hazard ratio (HR) and 95% confidence interval are also displayed below the *x*-axis. A total of 70 patients in the lymphopenia cohort and 57 patients in the no-lymphopenia cohort were excluded from this analysis because they experienced death before the start of the analysis window. Survival estimates beyond 24 months are based on Kaplan–Meier extrapolation with substantial right censoring and should be interpreted as exploratory; 36-month and 5-year estimates should therefore be considered as hypothesis-generating given the observed median follow-up of 105–123 days across cohorts.

Among patients included after propensity score matching (5249 per cohort), the primary outcome of OS was lower in the lymphopenia group. The mortality risk ratio (RR) was 1.16 with a 95% CI of 1.12–1.2. KM survival analysis, excluding patients with outcomes before the time window, showed a significant difference (*p* < 0.001). Cox analysis showed an HR of 1.26 (95% CI 1.2–1.33). The 24-month OS was 27.9% vs. 35.3%, respectively (Table 3 and Figure 2).

### 3.4. Secondary Outcomes

#### 3.4.1. Overall Survival at 6 Months

The 6-month OS was lower in the lymphopenia group than in the non-lymphopenia group. The mortality RR was 1.21 with a 95% CI of 1.16–1.26. KM survival analysis, excluding patients with outcomes before the time window, showed a significant difference (*p* < 0.001). Cox analysis showed an HR of 1.29 (95% CI 1.22–1.37). The 6-month OS was approximately 41.6% and 50.7%, respectively (Table 3 and Figure 2).

#### 3.4.2. Overall Survival at 12 Months

The 12-month OS was lower in the lymphopenia group than in the non-lymphopenia group. The mortality RR was 1.18 with a 95% CI of 1.14–1.23. KM survival analysis, excluding patients with outcomes before the time window, showed a significant difference (*p* < 0.001). Cox analysis showed an HR of 1.28 (95% CI 1.21–1.35). The 12-month OS was approximately 34.0% and 42.2%, respectively (Table 3 and Figure 2).

#### 3.4.3. Healthcare Utilization at 6 Months

**Figure 3:**
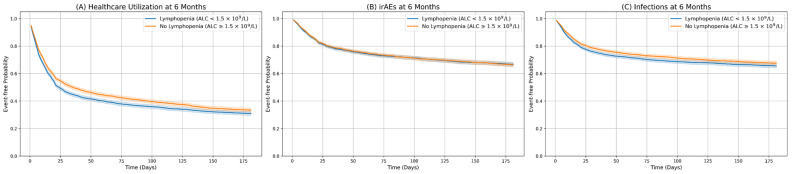
Kaplan–Meier curves for healthcare utilization, immune-related adverse events, and infections within 6 months of ICI initiation. Kaplan–Meier curves depicting time to first (**A**) healthcare utilization event, (**B**) immune-related adverse event (irAE), and (**C**) clinically coded infection within 6 months of immune checkpoint inhibitor (ICI) initiation, stratified by baseline lymphopenia status. Curves are shown for patients with baseline lymphopenia (blue line) and without baseline lymphopenia (orange line). IrAEs and infections were identified using prespecified ICD-10 diagnosis codes, and comparisons between groups were performed using log-rank tests. These analyses should be interpreted with caution, as they may be influenced by differential follow-up time and the competing risk of early mortality in the lymphopenic cohort; formal competing-risk analyses were not feasible within the TriNetX platform.

Baseline lymphopenia was associated with greater healthcare utilization. The RR was 1.05 (95% CI 1.02–1.09). KM analysis showed a significant difference (*p* < 0.001). Cox analysis showed an HR of 1.12 (95% CI 1.06–1.17). The 6-month event-free probability was approximately 31.0% and 33.4%, respectively (Table 4 and Figure 3).

#### 3.4.4. Immune-Related Adverse Events at 6 Months

Baseline lymphopenia was associated with a similar risk of irAEs. The RR was 1.0, 95% CI 0.93–1.06). KM analysis showed a *p*-value of 0.86. Cox analysis showed an HR of 1.0 (95% CI 0.93–1.08). The 6-month event-free probability was approximately 66.7% and 66.4%, respectively (Table 4 and Figure 3).

#### 3.4.5. Infections at 6 Months

Baseline lymphopenia was associated with increased infections. The RR was 1.08 (95% CI 1.01–1.15). KM analysis showed a significant difference (*p* = 0.006). Cox analysis showed an HR of 1.11 (95% CI 1.03–1.19). The 6-month event-free probability was approximately 65.6% and 67.6%, respectively (Table 4 and Figure 3).

### 3.5. Exploratory Overall Survival Outcomes

#### 3.5.1. Overall Survival at 36 Months

In this pre-specified exploratory analysis, which is subject to substantial right censoring given the observed median follow-up of 105–123 days, the 36-month OS was lower in the lymphopenia group than in the non-lymphopenia group. The mortality RR was 1.16 with a 95% CI of 1.12–1.2. KM survival analysis, excluding patients with outcomes before the time window, showed a significant difference (*p* < 0.001). Cox analysis showed an HR of 1.26 (95% CI 1.2–1.33). The 36-month OS was approximately 25.2% and 32.3%, respectively (Table 3 and Figure 2). These estimates should be considered hypothesis-generating only and require prospective validation.

#### 3.5.2. Overall Survival at 5 Years

In this pre-specified exploratory analysis, which is subject to substantial right censoring given the observed median follow-up of 105–123 days, the 5-year OS was lower in the lymphopenia group than in the non-lymphopenia group. The mortality RR was 1.15 with a 95% CI of 1.11–1.19. KM survival analysis excluding patients who died before the outcome window showed a significant difference (*p* < 0.001). Cox analysis showed an HR of 1.26 (95% CI 1.2–1.33). The 5-year OS was approximately 21.6% and 27.8%, respectively (Table 3 and Figure 2). These estimates should be considered hypothesis-generating only and require prospective validation.

#### 3.5.3. Overall Survival at 24 Months with ALC Measured 7 Days Before ICI Initiation

To explore the results of OS if ALC were measured 7 days before ICI initiation rather than within the 30-day window, we built two cohorts using the exact definitions described in our methods section for the lymphopenia and no-lymphopenia groups. Among patients included after propensity score matching (3515 per cohort), the primary outcome of OS was similarly lower in the lymphopenia group. The mortality RR was 1.25 with a 95% CI of 1.2–1.31. KM survival analysis, excluding patients with outcomes before the time window, showed a significant difference (*p* < 0.001). Cox analysis showed an HR of 1.38 (95% CI 1.29–1.47). The 24-month OS was 28.2% vs. 38.8%, respectively.

#### 3.5.4. Overall Survival at 24 Months in the Lymphopenia Cohort, Stratified by PD-1 Versus PD-L1

We built separate sub-groups using the exact characteristics used to build the lymphopenia group as previously mentioned in the methods section. The lymphopenia patients were categorized into two groups: PD-1 group, which included patients treated with pembrolizumab or nivolumab, and PD-L1 group, which included patients treated with atezolizumab. After propensity score matching (1262 per cohort), OS was similar in both groups. The mortality RR was 0.99 with a 95% CI of 0.93–1.05. KM survival analysis, excluding patients with the outcome prior to the time window, showed no significant difference (*p* = 0.27). Cox analysis showed an HR of 1.01 (95% CI 0.91–1.11). The 24-month OS was 23.9% vs. 22.6%, respectively.

### 3.6. Unmatched Outcomes for the Primary and Secondary Endpoints

To contextualize the propensity score-matched findings and assess the robustness of the primary and secondary results, we present the corresponding outcomes in the unmatched cohorts (lymphopenia, N = 10,837; no lymphopenia, N = 5303). Baseline characteristics of the unmatched cohorts differed across several covariates, most notably prior radiation history and antineoplastic use (standardized differences > 0.10), as detailed in Table 1. Despite these differences, the direction and magnitude of the association between baseline lymphopenia and overall survival remained consistent with the matched analysis, supporting the stability of the observed effect. The non-mortality secondary outcomes also showed a similar direction and magnitude of the association. Unmatched outcome estimates should be interpreted with caution, given the potential for residual confounding (Table 5).

### 3.7. Sub-Group Analysis

#### 3.7.1. Overall Survival at 24 Months Stratified by Major Tumor Sub-Groups

We built separate sub-groups using the exact characteristics used to build the lymphopenia and no-lymphopenia groups as previously mentioned in the Methods section based on the major tumor groups with adequate representation, allowing us to capture all patients that can be matched using the PSM model. The results showed that baseline lymphopenia remained associated with inferior OS across each subgroup, with hazard ratios of similar magnitude to those in the overall cohort, although confidence intervals were wider due to reduced sample sizes.

##### Gastrointestinal Cancers

**Figure 4:**
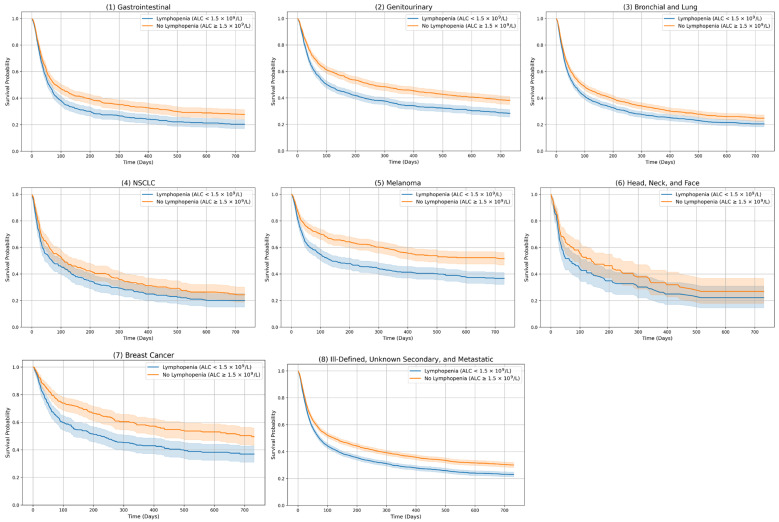
Kaplan–Meier Overall Survival Curves Stratified by Baseline Lymphopenia Status across Solid Tumor Subgroups. Kaplan–Meier curves depicting overall survival stratified by baseline lymphopenia status across solid tumor subtypes: (**1**) Gastrointestinal, (**2**) Genitourinary, (**3**) Bronchial and Lung, (**4**) Non-Small Cell Lung Cancer (NSCLC), (**5**) Melanoma, (**6**) Head, Neck, and Face, (**7**) Breast, and (**8**) Ill-defined, Unknown Secondary, and Metastatic cancers. All patients received immune checkpoint inhibitor (ICI) therapy and were identified from TriNetX. Cohorts were stratified by baseline absolute lymphocyte count (ALC): Lymphopenia (ALC < 1.5 × 10^9^/L; blue) versus No Lymphopenia (ALC ≥ 1.5 × 10^9^/L; orange). Shaded areas represent 95% confidence intervals. The *x*-axis denotes time from ICI initiation in days (range 0–730), and the *y*-axis denotes the Kaplan–Meier estimated survival probability. Across all tumor types, the lymphopenic cohort demonstrated a consistently lower survival probability than the non-lymphopenic cohort.

After propensity score matching (922 per cohort), OS was lower in the lymphopenia group. The mortality RR was 1.11 with a 95% CI of 1.03–1.19. KM survival analysis, excluding patients with the outcome prior to the time window, showed a significant difference (*p* < 0.001). Cox analysis showed an HR of 1.25 (95% CI 1.11–1.4). The 24-month OS was 20.3% vs. 27.7%, respectively (Table 6 and Figure 4).

##### Genitourinary Cancers

After propensity score matching (1653 per cohort), OS was lower in the lymphopenia group. The mortality RR was 1.22 with a 95% CI of 1.14–1.3. KM survival analysis, excluding patients with the outcome prior to the time window, showed a significant difference (*p* < 0.001). Cox analysis showed an HR of 1.36 (95% CI 1.2–1.5). The 24-month OS was 28.4% vs. 38.2%, respectively (Table 6 and Figure 4).

##### Bronchial and Lung Cancer

After propensity score matching (1996 per cohort), OS was lower in the lymphopenia group. The mortality RR was 1.08 with a 95% CI of 1.03–1.14. KM survival analysis, excluding patients with the outcome prior to the time window, showed a significant difference (*p* < 0.001). Cox analysis showed an HR of 1.18 (95% CI 1.1–1.28). The 24-month OS was 20.5% vs. 24.8%, respectively (Table 6 and Figure 4).

##### Non-Small Cell Lung Cancer

After propensity score matching, and despite a much lower overall number (301 per cohort), OS trended downward in the lymphopenia group. The mortality RR was 1.08 with a 95% CI of 0.97–1.2. KM survival analysis, excluding patients with the outcome prior to the time window, showed a *p*-value of 0.053. Cox analysis showed an HR of 1.21 (95% CI 1.0–1.46). The 24-month OS was 20.0% vs. 24.3%, respectively (Table 6 and Figure 4). The lack of statistical significance likely reflects limited sample size rather than a true null effect, as the HR direction and magnitude (1.21) remain consistent with the overall cohort.

##### Melanoma

After propensity score matching (645 per cohort), OS was lower in the lymphopenia group. The mortality RR was 1.4 with a 95% CI of 1.23–1.58. KM survival analysis, excluding patients with the outcome prior to the time window, showed a significant difference (*p* < 0.001). Cox analysis showed an HR of 1.56 (95% CI 1.32–1.85). The 24-month OS was 36.6% vs. 51.6%, respectively (Table 6 and Figure 4).

##### Head, Neck, and Face Cancers

After propensity score matching, and despite a much lower overall number (165 per cohort), OS trended downward in the lymphopenia group. The mortality RR was 1.18 with a 95% CI of 0.98–1.42. KM survival analysis, excluding patients with the outcome prior to the time window, showed a *p*-value of 0.097. Cox analysis showed an HR of 1.28 (95% CI 0.96–1.71). The 24-month OS was 22.2% vs. 26.9%, respectively (Table 6 and Figure 4). The lack of statistical significance likely reflects limited sample size rather than a true null effect, as the HR direction and magnitude (1.28) remain consistent with the overall cohort.

##### Breast Cancer

After propensity score matching (412 per cohort), OS was lower in the lymphopenia group. The mortality RR was 1.38 with a 95% CI of 1.17–1.63. KM survival analysis, excluding patients with the outcome prior to the time window, showed a significant difference (*p* < 0.001). Cox analysis showed an HR of 1.54 (95% CI 1.24–1.91). The 24-month OS was 36.9% vs. 49.6%, respectively (Table 6 and Figure 4).

##### Ill-Defined, Unknown Secondary, and Metastatic Category

After propensity score matching (3657 per cohort), OS was lower in the lymphopenia group. The mortality RR was 1.12 with a 95% CI of 1.08–1.17. KM survival analysis, excluding patients with the outcome prior to the time window, showed a significant difference (*p* < 0.001). Cox analysis showed an HR of 1.23 (95% CI 1.16–1.31). The 24-month OS was 23.0% vs. 30.1%, respectively (Table 6 and Figure 4).

#### 3.7.2. Overall Survival at 24 Months Stratified by Prior Lines of Therapies

##### Prior Radiotherapy at Any Time Before ICI Initiation

**Figure 5:**
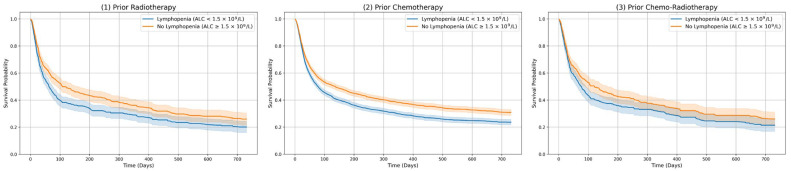
Kaplan–Meier Overall Survival Curves Stratified by Baseline Lymphopenia Status According to Prior Treatment Exposure. Kaplan–Meier curves depicting overall survival stratified by baseline lymphopenia status among patients with prior treatment exposure, including (**1**) Prior Radiotherapy, (**2**) Prior Chemotherapy, and (**3**) Prior Chemo-Radiotherapy. All patients received immune checkpoint inhibitor (ICI) therapy and were identified from TriNetX. Cohorts were stratified by baseline absolute lymphocyte count (ALC): Lymphopenia (ALC < 1.5 × 10^9^/L; blue) versus No Lymphopenia (ALC ≥ 1.5 × 10^9^/L; orange). Shaded areas represent 95% confidence intervals. The *x*-axis denotes time from ICI initiation in days (range 0–730), and the *y*-axis denotes the Kaplan–Meier estimated survival probability. Across all three prior treatment subgroups, patients with baseline lymphopenia demonstrated consistently inferior overall survival compared to those without lymphopenia, with the separation between curves most pronounced in the early follow-up period. These findings suggest that baseline lymphopenia retains its adverse prognostic significance regardless of the type of prior treatment received prior to ICI initiation.

After propensity score matching (626 per cohort), OS was lower in the lymphopenia group. The mortality RR was 1.12 with a 95% CI of 1.03–1.23. KM survival analysis, excluding patients with the outcome prior to the time window, showed a significant difference (*p* < 0.001). Cox analysis showed an HR of 1.28 (95% CI 1.11–1.48). The 24-month OS was 20.1% vs. 26.0%, respectively (Table 7 and Figure 5).

##### Prior Chemotherapy at Any Time Before ICI Initiation

After propensity score matching (2696 per cohort), OS was lower in the lymphopenia group. The mortality RR was 1.13 with a 95% CI of 1.08–1.18. KM survival analysis, excluding patients with the outcome prior to the time window, showed a significant difference (*p* < 0.001). Cox analysis showed an HR of 1.24 (95% CI 1.15–1.33). The 24-month OS was 23.6% vs. 30.8%, respectively (Table 7 and Figure 5).

##### Prior Chemo-Radiotherapy at Any Time Before ICI Initiation

After propensity score matching, and despite a much lower overall number (493 per cohort), OS trended downward in the lymphopenia group. The mortality RR was 1.08 with a 95% CI of 0.98–1.2. KM survival analysis, excluding patients with the outcome prior to the time window, showed a small significant difference with a *p*-value of 0.03. Cox analysis showed an HR of 1.19 (95% CI 1.02–1.4). The 24-month OS was 21.4% vs. 26.0%, respectively (Table 7 and Figure 5).

#### 3.7.3. Immune-Related Adverse Events at 6 Months Stratified by irAE Category

##### Gastrointestinal irAEs

After propensity score matching, The RR of GI irAEs was 0.98 with a 95% CI of 0.88–1.1. KM survival analysis showed a *p*-value of 0.91. Cox analysis showed an HR of 0.99 (95% CI 0.88–1.12). The 6-month event-free probability was 87.2% vs. 87.0%, respectively.

##### Pulmonary irAEs

After propensity score matching, The RR of pulmonary irAEs was 1.1 with a 95% CI of 0.84–1.42. KM survival analysis showed a *p*-value of 0.45. Cox analysis showed an HR of 1.11 (95% CI 0.85–1.44). The 6-month event-free probability was 97.2% vs. 97.5%, respectively.

##### Endocrinology irAEs

After propensity score matching, The RR of pulmonary irAEs was 1.0 with a 95% CI of 0.91–1.1. KM survival analysis showed a *p*-value of 0.82. Cox analysis showed an HR of 1.11 (95% CI 0.91–1.12). The 6-month event-free probability was 80.5% vs. 80.2%, respectively.

##### Dermatologic irAEs

After propensity score matching, The RR of pulmonary irAEs was 0.94 with a 95% CI of 0.76–1.16. KM survival analysis showed a *p*-value of 0.69. Cox analysis showed an HR of 0.96 (95% CI 0.77–1.19). The 6-month event-free probability was 95.7% vs. 95.6%, respectively.

## 4. Discussion

In this large, multi-center real-world cohort of a total identified population of 16,140, including 10,498 patients (5249 per group) of propensity-matched patients with diverse solid tumors with different stages treated with PD-1/PD-L1 inhibitors, baseline lymphopenia was consistently associated with inferior OS across 6-month, 12-month, and 24-month, besides the exploratory 36-month, and 5-year follow-up windows. The magnitude of the association was remarkably stable across the primary and secondary endpoints (6, 12, and 24 months), with HRs ranging from approximately 1.26 to 1.29 and an absolute 6–9 percentage-point decrement in OS over time. Such results suggest that low baseline ALC < 1.5 × 109/L may capture a durable vulnerability that persists well beyond the early treatment period. The directional consistency of the exploratory 36-month and 5-year estimates is noted; however, given the observed median follow-up of 105–123 days and the substantial right censoring underlying these projections, they cannot be taken as confirmatory evidence of a persistent long-term effect and should be interpreted as hypothesis-generating. These findings extend prior work on lymphocyte biology and ICI response by demonstrating that a single, clinically accessible ALC threshold may be prognostic across solid tumor types in routine practice.

Our findings suggest that baseline lymphopenia in ICI-treated patients with solid tumors operates along two clinically distinct axes simultaneously. The first is prognostic: patients with ALC < 1.5 × 10^9^/L had consistently inferior OS across all time horizons, with hazard ratios of 1.26–1.29 and an absolute survival decrement of 6–9 percentage points. Such HR was stable from 6 months through the primary outcome, 24 months (besides the exploratory outcomes, 36 months and 5 years), suggesting that baseline lymphopenia reflects a durable vulnerability rather than a transient immunologic state. The second is more provocative: despite this survival disadvantage and greater infectious complications, lymphopenic patients developed clinically coded irAEs at an identical rate to their non-lymphopenic counterparts (RR 1.0, 95% CI 0.93–1.06). This dissociation, between impaired immune reserve on the one hand and preserved capacity for immune-mediated toxicity on the other, is not readily explained by frailty or disease burden alone and challenges the assumption that immune competence and irAE risk are uniformly coupled along a single activation continuum. These findings could support the use of baseline ALC as a biomarker that informs not only survival prognosis but potentially the anticipated toxicity profile of ICI therapy, with distinct clinical implications along each axis. However, the irAE results should be interpreted with caution as such an observation is limited by EHR under-ascertainment, variable coding practices, shorter at-risk time, and unmodeled competing risk of early death.

### 4.1. Comparison with Prior Literature and Reconciliation of Conflicting Evidence

A substantial body of literature has established that lymphopenia, whether treatment-related or spontaneous, is common in cancer and portends worse outcomes, with severe lymphopenia in chemoradiation-treated populations consistently associated with reduced survival, likely reflecting both impaired antitumor immunity and increased susceptibility to infection and organ failure [9,10,14,23,24,25]. Meta-analytic data in tumors such as esophageal and lung cancers suggest that treatment-related lymphopenia during concurrent chemoradiation or combined-modality therapy is associated with inferior efficacy of subsequent or concurrent ICIs, underscoring the importance of preserving lymphocyte reserves for optimal checkpoint blockade activity [9,26,27].

In the ICI setting specifically, several retrospective series have demonstrated that lymphocyte dynamics after treatment initiation carry strong prognostic information [13,19,28]. Prior series have generally found on-treatment lymphocyte dynamics to be more strongly prognostic than baseline ALC alone, potentially because smaller sample sizes were underpowered to detect the modest but consistent hazard ratios (1.26–1.29) we observe here, or because lower ALC thresholds (e.g., <1.0 × 10^9^/L) were used that captured only the most profoundly immunosuppressed patients. Taken together with prior reports showing that on-treatment lymphocyte dynamics carry strong prognostic information, our findings suggest that baseline ALC may complement, rather than simply duplicate, the information provided by dynamic ALC measures reported in earlier studies.

Some studies have reported that baseline lymphopenia does carry prognostic weight in at least some ICI-treated populations and have suggested that low baseline ALC may also associate with worse outcomes [16,18]. A large real-world analysis of 18,186 metastatic solid tumor outpatients treated with ICIs showed that baseline blood cell counts, including lymphocyte parameters and composite indices such as NLR, correlate with survival across tumor types and treatment settings [16]. Notably, in metastatic melanoma, development of lymphopenia during PD-1/CTLA-4 blockade has been linked to shorter PFS and OS [28,29]. Collectively, these data support lymphocyte metrics as prognostic in the ICI era, yet whether a single pretreatment ALC threshold is sufficient for risk stratification remains unclear.

Our study helps resolve this uncertainty by demonstrating that, in a broad pan-cancer real-world cohort with rigorous propensity matching, a prespecified baseline ALC cutoff (<1.5 × 10^9^/L vs. ≥1.5 × 10^9^/L) is reproducibly associated with worse OS, with the effect evident as early as 6 months. Unlike prior single-center or tumor-restricted analyses [17,19], we observed consistent hazard ratios 1.26–1.29 across all time points, with Kaplan–Meier curves diverging early at 6 and 12 months and remaining separated at 24 months besides the exploratory 36 months and 5 years. These findings remained consistent whether ALC was measured 30 days or even on a narrower scale, 7 days, before ICI initiation. They also remained evident in all the subgroup analyses, whether through stratification using the tumor type or by previous lines of therapy, suggesting that baseline lymphopenia reflects a stable risk state rather than transient noise in different types of solid tumors. These findings align with population-based data outside the ICI context, showing that lymphopenia in the general population is associated with markedly increased long-term all-cause mortality [11,12,30], supporting the concept that low lymphocyte counts reflect impaired immune reserve and frailty. By anchoring our analysis in a large, heterogeneous real-world dataset, we provide complementary evidence that baseline ALC alone, before any treatment-related changes, may serve as a meaningful prognostic biomarker in ICI-treated patients with solid tumors.

Several design differences may explain why our results diverge from the negative or equivocal findings reported in the literature [17,19,31]. For example, Sun et al. found that baseline lymphopenia did not consistently predict survival in early-phase ICI trials, whereas on-treatment lymphocyte dynamics were more informative [31]. Conroy et al. also reported limited prognostic value of baseline ALC in a smaller pan-cancer cohort treated with ICIs, with hazard ratios closer to 1.0 [17]. Our study is substantially larger and more heterogeneous, including 10,498 matched patients across multiple tumor types, practice settings, and lines of therapy, thereby increasing power to detect modest but clinically meaningful hazard ratios of around 1.2–1.3. Additionally, we used a slightly higher, more clinically intuitive ALC cutoff (1.5 × 10^9^/L) that approximates the lower limit of normal in many laboratories. In contrast, some prior studies used lower thresholds (e.g., 1.0 × 10^9^/L), potentially reporting milder immunologic deficits as “normal” [17]. Furthermore, we applied extensive propensity score matching across a wide set of demographic, cancer-related, comorbidity, and treatment variables, thereby reducing confounding by factors such as tumor burden, prior therapy, and radiation exposure, which are known to influence ALC and ICI outcomes. Smaller, single-center studies with more limited adjustment may have been underpowered or more susceptible to residual confounding, especially when baseline lymphopenia is correlated with other poor-prognosis features. Finally, the endpoints differ. Some early work focused primarily on radiographic response rates rather than survival. In contrast, our primary endpoint was OS at 24 months, capturing both direct treatment effects and the broader impact of immune competence on long-term outcomes.

Our findings should therefore not be interpreted as contradicting the importance of on-treatment lymphopenia. Instead, they suggest that baseline and dynamic ALC may provide complementary information. Baseline lymphopenia appears to identify a high-risk subgroup before treatment begins. In contrast, the development or persistence of lymphopenia during therapy captures additional prognostic information about treatment tolerance and evolving disease biology. Future work integrating baseline ALC, early on-treatment changes, and other hematologic indices (e.g., NLR, platelet-to-lymphocyte ratio) may yield even more powerful prognostic models for ICI-treated patients.

### 4.2. Relationship to Immune-Related Adverse Events and Infections

The relationship between lymphocyte counts and irAEs has also been an area of active investigation. Several studies suggest that higher baseline lymphocyte counts or lower NLR may be associated with a higher risk of irAEs, potentially reflecting a more reactive immune reservoir [32,33,34]. A multicenter NSCLC cohort showed that baseline ALC and early changes in ALC were associated with the incidence of irAEs under nivolumab monotherapy, with patients who developed irAEs tending to have more favorable immune profiles and better survival [20]. Another study found that irAEs, particularly cutaneous and endocrine events, are often associated with improved outcomes, reinforcing the idea that vigorous immune activation is a double-edged sword that can generate both toxicity and antitumor benefit [35]. The relationship between baseline lymphopenia and irAEs is the most analytically complex and clinically instructive finding of this study. As reported in our study, baseline lymphopenia was not associated with a higher incidence of clinically coded irAEs at 6 months (RR 1.0). The prevailing framework in ICI biomarker research suggests that patients with more robust immune status generate both better antitumor responses and greater immune-mediated toxicity, whereas those with suppressed immune function experience both treatment failure and relative protection from toxicity [32,35].

At least three mechanistic explanations deserve consideration, each with distinct clinical implications. First, competing mortality may obscure a true protective signal. Lymphopenic patients died at higher rates early, accruing less exposure time during which irAEs could develop and be coded. A formal competing risk analysis using sub-distribution hazard methods would be required to determine whether the observed RR of 1.0 underestimates a true biological difference in irAE susceptibility. Second, the biology may be genuine. Patients with depleted circulating lymphocyte reserves may lack the effector capacity to mount the autoimmune responses that underlie irAEs, consistent with prior observations that irAEs correlate with more favorable baseline immune profiles and that vigorous immune activation is necessary for both antitumor efficacy and off-target toxicity [33,36]. Third, ICD-10-based irAE ascertainment is subject to misclassification in sicker patients, in whom inflammatory complications may be coded to alternative diagnostic categories such as infection or respiratory failure. The convergence of these explanations around a single, clinically actionable implication is notable: baseline ALC cannot reassure clinicians that a lymphopenic patient carries lower irAE risk, even as it reliably identifies elevated mortality risk. These are distinct clinical signals requiring separate assessment and management frameworks.

At the same time, we observed a higher 6-month infection risk in the lymphopenia cohort (RR 1.08, 95% CI 1.01–1.15). This is consistent with the well-established association between lymphopenia and infectious complications in both oncology and general populations [9,36,37]. The combination of worse survival, greater healthcare utilization, and a possible increase in serious infections, without a clear increase in irAEs, suggests a model in which baseline lymphopenia marks patients who may be less able to tolerate disease and treatment-related stressors rather than those at particular risk for immune overactivation.

### 4.3. Clinical Implications

Consistent with this model, our results support incorporating baseline ALC into routine risk assessment for patients with solid tumors being considered for ICI therapy. A simple binary threshold of <1.5 × 10^9^/L versus ≥1.5 × 10^9^/L, which is available in virtually all practice settings, may help identify patients with approximately up to a 29% higher relative hazard of death and lower survival rates across multiple time horizons, even after adjusting for tumor type, metastatic status, prior treatments, and key comorbidities. Importantly, the dissociation between mortality risk and irAE risk means that baseline lymphopenia should inform two distinct clinical decisions independently. For mortality risk: more intensive monitoring, lower thresholds for evaluating new symptoms, proactive infection prevention, and early supportive care integration. For irAE risk: lymphopenic status alone should not be used to reduce vigilance for immune toxicity. The preserved irAE rate in this population, whether reflecting genuine biological equivalence or competing mortality, argues against assuming relative safety from immune-related complications based on ALC.

In parallel, our findings suggest that clinical trials of ICIs and combination regimens should record and report baseline ALC and lymphocyte dynamics and consider stratifying or adjusting randomization by ALC to avoid imbalances in immune reserves that could confound treatment effect estimates. The absence of a differential survival effect between anti-PD-1 and anti-PD-L1 agents within the lymphopenic cohort further supports the interpretation that baseline immune reserve, rather than the specific checkpoint targeted, drives the observed survival disadvantage. Notably, composite inflammatory scores such as the Lung Immune Prognostic Index (LIPI), which incorporates derived neutrophil-to-lymphocyte ratio and lactate dehydrogenase level, have demonstrated similar prognostic value in ICI-treated patients [38]; our findings suggest that integrating baseline ALC with such multi-marker indices may enhance risk stratification beyond what either parameter achieves alone.

Our findings also raise an important and clinically actionable question: if baseline lymphopenia identifies patients unlikely to derive full benefit from ICI therapy because of higher mortality risks, can targeted lymphocyte reconstitution before or alongside ICI initiation improve outcomes? Preclinical evidence suggests that this is a tractable strategy. T-lymphopenia has been shown to impair anti-PD-1 efficacy by reducing the pool of tumor-reactive PD-1^+^ CD8 T cells within the tumor microenvironment, without necessarily reducing total tumor-infiltrating lymphocyte numbers, a mechanistic dissociation that makes ALC a particularly relevant systemic biomarker [39,40,41]. In murine models, administration of recombinant long-acting IL-7 (rhIL-7-hyFc; efineptakin alfa, NT-I7) selectively restored PD-1^+^ CD8 tumor-infiltrating lymphocyte populations, expanded stem-like progenitor T cells, and significantly improved tumor regression and survival when combined with anti-PD-1 therapy in severely lymphopenic animals [39,42]. Clinically, IL-7 has been shown to safely and durably increase ALC in lymphopenic cancer patients [43,44,45]. In the phase IIa ELYPSE-7 trial, CYT107 (recombinant IL-7) administered before chemotherapy produced a 148% increase in CD4^+^ T-cell counts versus 9.9% in the placebo group in lymphopenic patients with metastatic breast cancer (*p* = 0.002) and was well tolerated [44]. The long-acting IL-7 fusion protein efineptakin alfa (NT-I7) is currently being evaluated in combination with pembrolizumab in a Phase 1b/2a basket trial (NCT04332653) across multiple advanced solid tumor types including triple-negative breast cancer, NSCLC, and colorectal cancer, with lymphocyte expansion as a key pharmacodynamic endpoint [46]. Similarly, IL-15-based immunocytokines, including the superagonist N-803 (Anktiva), have demonstrated robust NK-cell and CD8^+^ T-cell expansion and are being studied in combination with PD-1/PD-L1 blockade in solid tumors [47,48,49]. Beyond cytokine-based approaches, bispecific T-cell engagers (BiTEs) and related immunocytokine platforms capable of redirecting and locally expanding T cells represent complementary strategies that could, in principle, overcome the deficient T-cell pool that characterizes lymphopenic patients prior to ICI initiation [50,51,52]. Whether pre-treating lymphopenic patients with systemic T-cell amplifying agents, such as long-acting IL-7 or IL-15 superagonists, prior to ICI initiation could restore the circulating immune reserve needed for checkpoint blockade efficacy is a hypothesis that our data directly supports and that merits prospective investigation.

### 4.4. Strengths and Limitations

This study has several notable strengths. It leverages the large, diverse US Collaborative Network within TriNetX, encompassing 67 health care organizations and both academic and community settings, which enhances the generalizability of our findings to real-world ICI practice. The study includes a broad spectrum of solid tumors and three widely used ICIs (pembrolizumab, nivolumab, atezolizumab), allowing a pan-cancer assessment rather than being only confined to a single tumor type. Rigorous propensity score matching across demographic, diagnostic, procedural, medication, and laboratory variables yielded well-balanced cohorts with standardized differences <0.1, reducing confounding by measured factors such as tumor site mix, comorbidities, prior radiation, and concomitant corticosteroid or immunosuppressant use. Additionally, we evaluated survival outcomes across multiple clinically relevant time points from 6, 12, and 24 months and up to an exploratory time window of 36 months and 5 years. We also incorporated healthcare utilization, irAEs, and serious infections, providing a comprehensive view of how baseline lymphopenia affects safety outcomes in ICI-treated patients. In addition to providing pan-solid cancer prognostic data, our study provides stratified data across various solid tumor types and prior lines of therapy, showing that OS outcomes remain consistent and stable, irrespective of solid tumor subtype or prior lines of therapy before ICI initiation.

Several limitations also warrant consideration. The most important methodological limitation is the short observed median follow-up of 105–123 days across all matched cohorts, which is inherent to the TriNetX platform, where follow-up is computed to the last recorded encounter rather than through active prospective surveillance. This could reflects high early mortality and the censoring structure of the TriNetX platform, which does not export individual censoring event data; consequently, the precise number of patients censored at each time point could not be reported. As a result, the 36-month and 5-year Kaplan–Meier estimates are based on extensive right censoring and carry substantially greater uncertainty than their confidence intervals convey. These long-term estimates are pre-specified exploratory endpoints and should not be interpreted as confirmatory evidence of a persistent survival disadvantage beyond 24 months. As an observational study using EHR-derived data, our analysis is subject to residual confounding by unmeasured factors, such as performance status, tumor burden, PD-L1 expression, tumor mutational burden, and detailed treatment sequencing, which are incompletely captured in TriNetX. In addition, detailed tumor stage at the time of ICI initiation was unavailable for most patients in TriNetX, limiting our ability to fully adjust for stage-specific prognosis. The EHR platform does not provide reliable, standardized information on the exact number or the duration of ICI cycles received across participating sites. Consequently, we could not stratify outcomes by treatment intensity or cumulative ICI exposure, and residual confounding by treatment duration is possible. Misclassification of exposures and outcomes is possible due to variability in coding practices, incomplete capture of outside care, and under-coding of irAEs, which may bias effect estimates toward the null, particularly for toxicity endpoints. Baseline ALC was defined using the most recent value within 30 days before ICI initiation; in some patients, this may not reflect steady-state immune status but rather transient fluctuations due to intercurrent illness or steroid use. Additionally, irAE rates as assessed by ICD-10 coding are subject to competing risk from early mortality, such that the observed RR of 1.0 may underestimate a true biological difference in irAE susceptibility. Formal competing risk modeling using cause-specific or subdistribution hazard approaches is an important direction for future work. Finally, we could not fully disentangle treatment-related from intrinsic host contributions to lymphopenia, despite adjusting for prior radiation and antineoplastic exposure, which limits causal interpretation.

## 5. Conclusions

In summary, this large, real-world propensity-matched analysis demonstrates that baseline lymphopenia stratifies ICI-treated patients with solid tumors along two partially independent clinical axes. First, a mortality risk axis, on which lymphopenic patients carry a consistently 26–29% higher relative hazard of death at 6, 12, and 24 months, with similar hazard ratios observed in exploratory 36-month and 5-year analyses. In addition, an immune activation axis, on which the rate of clinically coded irAEs, despite inferior survival, was as frequent in patients with baseline lymphopenia as in those without, although this observation is limited by EHR under-ascertainment, variable coding practices, shorter at-risk time, and unmodeled competing risk of early death. Such a dissociation challenges the assumption that immune competence and irAE susceptibility are uniformly coupled. These findings suggest integrating baseline ALC into routine risk stratification and trial design while highlighting the need for prospective studies that combine baseline and on-treatment lymphocyte metrics with molecular and clinical biomarkers to optimize ICI use.

## Figures and Tables

**Table 1:** Baseline Characteristics Before and After Propensity Score Matching.

Characteristic	Lymphopenia (<1.5 × 10^9^/L) Before PSM (N = 10,837)	No Lymphopenia (≥1.5 × 10^9^/L) Before PSM (N = 5303)	Std Diff.	Lymphopenia (<1.5 × 10^9^/L) After PSM (N = 5249)	No Lymphopenia (≥1.5 × 10^9^/L) After PSM (N = 5249)	Std Diff.
Age (TriNetX built-in)						
Age at index, years (mean ± SD)	67.0 ± 12.1	65.7 ± 12.8	0.103	65.6 ± 12.6	65.9 ± 12.6	0.025
Gender (TriNetX built-in)						
Female sex	4749 (43.8%)	2567 (48.4%)	0.092	2507 (47.8%)	2521 (48.0%)	0.005
Male sex	6082 (56.1%)	2733 (51.5%)	0.092	2740 (52.2%)	2725 (51.9%)	0.006
Race (TriNetX built-in)						
White	8378 (77.3%)	3939 (74.3%)	0.071	3860 (73.5%)	3911 (74.5%)	0.022
Black or African American	1138 (10.5%)	667 (12.6%)	0.065	677 (12.9%)	650 (12.4%)	0.015
Asian	440 (4.1%)	202 (3.8%)	0.013	209 (4.0%)	202 (3.8%)	0.007
Ethnicity (TriNetX built-in)						
Hispanic or Latino	512 (4.7%)	289 (5.4%)	0.033	294 (5.6%)	282 (5.4%)	0.010
Not Hispanic or Latino	8820 (81.4%)	4159 (78.4%)	0.074	4117 (78.4%)	4127 (78.6%)	0.005
Primary Tumor Site (ICD-10)						
Lip, oral cavity, pharynx (C00–C14)	828 (7.6%)	216 (4.1%)	0.152	194 (3.7%)	216 (4.1%)	0.022
Digestive organs (C15–C26)	2486 (22.9%)	934 (17.6%)	0.133	917 (17.5%)	934 (17.8%)	0.008
Respiratory/intrathoracic (C30–C39)	4635 (42.8%)	2063 (38.9%)	0.079	2132 (40.6%)	2061 (39.3%)	0.028
Bone/cartilage (C40–C41)	307 (2.8%)	118 (2.2%)	0.039	119 (2.3%)	118 (2.2%)	0.001
Melanoma/skin (C43–C44)	1866 (17.2%)	994 (18.7%)	0.040	937 (17.9%)	973 (18.5%)	0.018
Mesothelial/soft tissue (C45–C49)	644 (5.9%)	293 (5.5%)	0.018	291 (5.5%)	288 (5.5%)	0.003
Breast (C50)	751 (6.9%)	454 (8.6%)	0.061	442 (8.4%)	431 (8.2%)	0.008
Female genital (C51–C58)	989 (9.1%)	517 (9.7%)	0.021	513 (9.8%)	515 (9.8%)	0.001
Male genital (C60–C63)	730 (6.7%)	297 (5.6%)	0.047	313 (6.0%)	297 (5.7%)	0.013
Urinary tract (C64–C68)	1821 (16.8%)	1033 (19.5%)	0.069	1036 (19.7%)	1021 (19.5%)	0.007
Eye/brain/CNS (C69–C72)	444 (4.1%)	184 (3.5%)	0.033	175 (3.3%)	184 (3.5%)	0.009
Thyroid/endocrine (C73–C75)	211 (1.9%)	95 (1.8%)	0.011	104 (2.0%)	94 (1.8%)	0.014
Ill-defined/secondary (C76–C80)	7912 (73.0%)	3589 (67.7%)	0.117	3657 (69.7%)	3575 (68.1%)	0.034
Key Comorbidities (ICD-10)						
Diabetes mellitus (E08–E13)	2788 (25.7%)	1417 (26.7%)	0.023	1379 (26.3%)	1401 (26.7%)	0.009
Chronic kidney disease (N18)	1943 (17.9%)	859 (16.2%)	0.046	892 (17.0%)	857 (16.3%)	0.018
Alcoholic liver disease (K70)	229 (2.1%)	73 (1.4%)	0.056	74 (1.4%)	73 (1.4%)	0.002
HIV disease (B20)	66 (0.6%)	43 (0.8%)	0.024	45 (0.9%)	41 (0.8%)	0.008
Inflammatory polyarthropathies (M05–M14)	1343 (12.4%)	596 (11.2%)	0.036	597 (11.4%)	595 (11.3%)	0.001
Heart failure (I50)	1560 (14.4%)	624 (11.8%)	0.078	624 (11.9%)	624 (11.9%)	<0.001
COPD (J44)	2605 (24.0%)	1179 (22.2%)	0.043	1226 (23.4%)	1177 (22.4%)	0.022
Hypertensive diseases (I10–I1A)	6924 (63.9%)	3270 (61.7%)	0.046	3264 (62.2%)	3248 (61.9%)	0.006
Peripheral arterial disease (I70–I79)	2701 (24.9%)	1186 (22.4%)	0.060	1203 (22.9%)	1183 (22.5%)	0.009
Cerebrovascular disease (I60–I69)	2011 (18.6%)	947 (17.9%)	0.018	988 (18.8%)	941 (17.9%)	0.023
Degenerative CNS diseases (G30–G32)	381 (3.5%)	174 (3.3%)	0.013	176 (3.4%)	173 (3.3%)	0.003
Medications & Previous Management Lines						
Personal history of irradiation (Z92.3)	2266 (20.9%)	632 (11.9%)	0.245	649 (12.4%)	632 (12.0%)	0.010
Antineoplastic agents (L01)	5517 (50.9%)	2263 (42.7%)	0.166	2298 (43.8%)	2258 (43.0%)	0.015
Glucocorticoids (H02AB)	8860 (81.8%)	4081 (77.0%)	0.119	4091 (77.9%)	4051 (77.2%)	0.018
Immunosuppressants (L04A)	480 (4.4%)	218 (4.1%)	0.016	212 (4.0%)	218 (4.2%)	0.006
Antibiotics (A07AA) ^a^	4123 (38.0%)	1776 (33.5%)	0.095	1867 (35.6%)	1772 (33.8%)	0.038
Proton pump inhibitors (A02BC) ^a^	6010 (55.5%)	2685 (50.6%)	0.097	2776 (52.9%)	2673 (50.9%)	0.039
Laboratory values ^a^						
Leukocytes, 10^9^/L	13.4 ± 136.7	30.3 ± 294.7	0.073	13.9 ± 128.6	30.5 ± 296.1	0.072
LDH, U/L	365.5 ± 576.8	370.3 ± 610.7	0.008	394.4 ± 634.8	371.2 ± 612.1	0.037
Creatinine, mg/dL	1.0 ± 0.7	1.0 ± 0.8	0.002	1.0 ± 0.8	1.0 ± 0.8	0.011
Neutrophils, 10^9^/L	15.4 ± 206.7	58.3 ± 543.3	0.104	19.7 ± 249.6	58.7 ± 545.6	0.092

The table shows baseline characteristics of both cohorts. Patients may carry multiple ICD codes. Leukocyte count, neutrophil count, antibiotic use, and proton pump inhibitor use were not included in propensity score matching: laboratory values were excluded to avoid over-adjustment for variables that are biologically correlated with the exposure (ALC), and medications were excluded as potential mediators of disease severity rather than independent confounders. The large standard deviations observed for leukocyte and neutrophil counts reflect known data-entry heterogeneity across contributing sites in the TriNetX network, including variability in units of measurement (e.g., cells/μL vs. ×10^9^/L), extreme outlier values from automated laboratory interfaces, and heterogeneous reporting conventions across 67 participating institutions. These variables were not included in the propensity score model and did not influence the matched cohort analyses. PSM, propensity score matching; Std diff., standardized difference; ICD-10, International Statistical Classification of Diseases and Related Health Problems, 10th Revision; HIV, human immunodeficiency virus; COPD, chronic obstructive pulmonary disease; CNS, central nervous system; LDH, lactate dehydrogenase. ^a^ Not included in PSM.

**Table 2:** Follow-up Duration by Analysis Time Window Before and After Propensity Score Matching.

	Before PSM, Lymphopenia			Before PSM, No Lymphopenia			After PSM, Lymphopenia			After PSM, No Lymphopenia		
Time Window	Mean ± SD (Days)	Median (Days)	IQR (Days)	Mean ± SD (Days)	Median (Days)	IQR (Days)	Mean ± SD (Days)	Median (Days)	IQR (Days)	Mean ± SD (Days)	Median (Days)	IQR (Days)
6 months (secondary outcome)	106.5 ± 68.8	105	144	110.2 ± 70.2	124	143	105.7 ± 69.4	105	146	110.0 ± 70.2	123	143
12 months (secondary outcome)	163.6 ± 140.4	105	329	174.0 ± 144.2	124	328	162.7 ± 141.0	105	331	173.6 ± 144.2	123	328
24 months (primary outcome)	232.5 ± 256.4	105	330	252.4 ± 265.8	124	403	232.1 ± 257.5	105	335	251.8 ± 265.6	123	400
36 months (exploratory outcome)	275.7 ± 348.6	105	330	300.5 ± 361.2	124	403	275.7 ± 350.3	105	335	299.8 ± 361.0	123	400
5 years (exploratory outcome)	319.0 ± 466.0	105	330	349.3 ± 485.8	124	403	319.2 ± 467.9	105	335	348.7 ± 485.9	123	400

IQR, interquartile range; PSM, propensity score matching; SD, standard deviation. Follow-up was measured from the index date to death, last recorded healthcare encounter, or the end of the analysis window, whichever occurred first.

**Table 3:** Overall survival outcomes after propensity score matching.

	Risk Analysis					Kaplan–Meier Survival Analysis			
Time Window	Mortality Risk % Lymphopenia	Mortality Risk % No Lymphopenia	Risk Difference (95% CI)	RR (95% CI)	*p*-Value	OS % Lymphopenia	OS % No Lymphopenia	HR (95% CI)	*p*-Value (Log-Rank)
6 months (secondary outcome)	50.5%	41.6%	8.8% (6.9–10.7)	1.21 (1.16–1.26)	<0.001	41.6%	50.7%	1.29 (1.22–1.37)	<0.001
12 months (secondary outcome)	55.0%	46.6%	8.4% (6.5–10.3)	1.18 (1.14–1.23)	<0.001	34.0%	42.2%	1.28 (1.21–1.35)	<0.001
24 months (primary outcome)	57.8%	49.7%	8.0% (6.1–9.9)	1.16 (1.12–1.20)	<0.001	27.9%	35.3%	1.26 (1.20–1.33)	<0.001
36 months (exploratory outcome)	58.6%	50.7%	8.0% (6.1–9.9)	1.16 (1.12–1.20)	<0.001	25.2%	32.3%	1.26 (1.20–1.33)	<0.001
5 years (exploratory outcome)	59.2%	51.4%	7.9% (6.0–9.8)	1.15 (1.11–1.19)	<0.001	21.6%	27.8%	1.26 (1.20–1.33)	<0.001

Each cohort consisted of N = 5249. Risk difference is the absolute difference in mortality risk between the lymphopenia and no-lymphopenia cohorts at each time point. A total of 70 patients in the lymphopenia cohort and 57 patients in the no-lymphopenia cohort were excluded from each time-window analysis because they experienced the outcome (death) before the start of that window. OS = overall survival; HR = hazard ratio; RR = risk ratio; CI = confidence interval.

**Table 4:** Six-Month Non-Mortality Secondary Outcomes.

	Risk Analysis					Kaplan–Meier Analysis			
Outcome (6-Month Window)	Risk % Lymphopenia	Risk % No Lymphopenia	Risk Difference (95% CI)	RR (95% CI)	*p*-Value	Event-Free % Lymphopenia	Event-Free % No Lymphopenia	HR (95% CI)	*p*-Value (Log-Rank)
Healthcare utilization	60.1%	57.2%	3.0% (1.1–4.8)	1.05 (1.02–1.09)	0.002	31.0%	33.4%	1.12 (1.06–1.17)	<0.001
Immune-related adverse events (irAEs)	26.3%	26.4%	−0.1% (−1.8–1.6)	1.00 (0.93–1.06)	0.877	66.7%	66.4%	1.01 (0.93–1.09)	0.863
Infections	28.8%	26.7%	2.1% (0.4–3.8)	1.08 (1.01–1.15)	0.016	65.6%	67.6%	1.11 (1.03–1.19)	0.006

Each cohort consisted of N = 5249. Risk difference is the absolute difference in mortality risk between the lymphopenia and no-lymphopenia cohorts at each time point. All outcomes were evaluated during the first 6 months after initiation of immune checkpoint inhibitor therapy. Event-free percentages correspond to KM survival probabilities for remaining free of the specified outcome at 6 months. RR = risk ratio; HR = hazard ratio; CI = confidence interval.

**Table 5:** Unmatched Mortality and Non-Mortality Outcomes by Baseline Lymphopenia Status.

**Mortality Outcomes**									
	**Risk Analysis**					**Kaplan–Meier Survival Analysis**			
**Time** **Window**	**Mortality** **Risk %** **Lymphopenia**	**Mortality** **Risk %** **No Lymphopenia**	**Risk Difference** **(95% CI)**	**RR** **(95% CI)**	***p*-Value**	**OS %** **Lymphopenia**	**OS %** **No Lymphopenia**	**HR** **(95% CI)**	***p*-Value** **(Log-Rank)**
6 months (secondary outcome)	53.5%	42.7%	10.7% (9.0–12.5)	1.25 (1.21–1.30)	<0.0001	38.2%	49.3%	1.34 (1.27–1.41)	<0.0001
12 months (secondary outcome)	57.6%	46.3%	11.3% (9.7–13.0)	1.24 (1.20–1.29)	<0.0001	31.2%	42.6%	1.36 (1.30–1.43)	<0.0001
24 months (primary outcome)	60.4%	49.4%	11.1% (9.4–12.7)	1.22 (1.19–1.26)	<0.0001	25.0%	35.6%	1.35 (1.29–1.42)	<0.0001
36 months (exploratory outcome)	61.2%	50.3%	10.9% (9.2–12.5)	1.22 (1.18–1.25)	<0.0001	22.7%	32.6%	1.35 (1.29–1.41)	<0.0001
5 years (exploratory outcome)	61.8%	51.0%	10.8% (9.1–12.4)	1.21 (1.18–1.25)	<0.0001	19.2%	28.1%	1.35 (1.29–1.41)	<0.0001
**Non-Mortality Outcomes**									
	**Risk Analysis**					**Kaplan–Meier Analysis**			
**Outcome** **(6-Month Window)**	**Risk %** **Lymphopenia**	**Risk %** **No Lymphopenia**	**Risk Difference** **(95% CI)**	**RR** **(95% CI)**	***p*** **-Value**	**Event-Free %** **Lymphopenia**	**Event-Free %** **No Lymphopenia**	**HR** **(95% CI)**	***p*** **-Value** **(Log-Rank)**
Healthcare utilization	61.7%	58.1%	3.6% (1.9–5.3)	1.06 (1.03–1.09)	<0.0001	29.6%	32.4%	1.12 (1.07–1.17)	<0.0001
Immune-related adverse events (irAEs)	26.2%	26.4%	−0.2% (−1.7–1.3)	0.99 (0.94–1.05)	0.799	67.5%	66.4%	1.00 (0.93–1.07)	0.921
Infections	29.6%	28.0%	1.6% (0.003–3.1)	1.06 (1.00–1.12)	0.051	64.7%	66.0%	1.07 (1.00–1.14)	0.050

Lymphopenia N = 10,837 and No Lymphopenia N = 5303. For the mortality outcomes, 137 patients in the lymphopenia cohort and 58 in the no-lymphopenia cohort were excluded from each time-window mortality analysis because they experienced the outcome (death) before the start of the analysis window. All secondary outcomes were evaluated during the first 6 months after initiation of immune checkpoint inhibitor therapy in the unmatched cohorts (before propensity score matching). Event-free percentages represent KM survival probabilities for remaining free percentages of the specified outcome at 6 months. OS = overall survival; HR = hazard ratio; RR = risk ratio; CI = confidence interval.

**Table 6:** Twenty-Four–Month Overall Survival by Tumor Type After Propensity Score Matching.

	Risk Analysis (24-Month Mortality)					Kaplan–Meier Survival Analysis (24-Month OS)			
Tumor Type	Mortality Risk % Lymphopenia	Mortality Risk % No Lymphopenia	Risk Difference (95% CI)	RR (95% CI)	*p*-Value	OS % Lymphopenia	OS % No Lymphopenia	HR (95% CI)	*p*-Value (Log-Rank)
Gastrointestinal (GI)	64.5%	58.4%	6.2% (1.7–10.6)	1.11 (1.03–1.19)	0.007	20.3%	27.7%	1.25 (1.11–1.40)	0.0002
Genitourinary (GU)	56.4%	46.4%	10.0% (6.6–13.5)	1.22 (1.14–1.30)	<0.0001	28.4%	38.2%	1.36 (1.23–1.49)	<0.0001
Bronchial and lung	65.5%	60.4%	5.1% (2.1–8.1)	1.08 (1.03–1.14)	0.0009	20.5%	24.8%	1.18 (1.10–1.28)	<0.0001
Non–small cell lung cancer (NSCLC)	72.1%	67.0%	5.1% (−2.2–12.5)	1.08 (0.97–1.20)	0.1714	20.0%	24.3%	1.21 (1.00–1.46)	0.0533
Melanoma	52.5%	37.8%	14.8% (9.4–20.2)	1.39 (1.23–1.58)	<0.0001	36.6%	51.6%	1.56 (1.32–1.85)	<0.0001
Head, neck, and face	63.1%	53.5%	9.6% (−1.2–20.4)	1.18 (0.98–1.42)	0.0837	22.2%	26.9%	1.28 (0.96–1.71)	0.0976
Breast	48.3%	35.0%	13.2% (6.5–19.9)	1.38 (1.17–1.63)	0.0001	36.9%	49.6%	1.54 (1.24–1.91)	<0.0001
Ill-defined/secondary/metastatic	61.8%	55.0%	6.8% (4.5–9.0)	1.12 (1.08–1.17)	<0.0001	23.0%	30.1%	1.23 (1.16–1.31)	<0.0001

For each tumor-type subgroup, patients with death prior to the start of the 24-month analysis window were excluded from risk and KM analyses (GI: 10 vs. 10; GU: 25 vs. 22; bronchial and lung: 27 vs. 18; NSCLC: 10 vs. 10; melanoma: 15 vs. 10; head/neck/face: 10 vs. 10; breast: 10 vs. 10; ill-defined/secondary/metastatic: 58 vs. 51 in lymphopenia vs. no-lymphopenia cohorts, respectively). OS = overall survival; HR = hazard ratio; RR = risk ratio; CI = confidence interval.

**Table 7:** Twenty-Four–Month Overall Survival by Prior Lines of Therapy After Propensity Score Matching.

	Risk Analysis (24-Month Mortality)					Kaplan–Meier Survival Analysis (24-Month OS)			
Prior Therapy	Mortality Risk % Lymphopenia	Mortality Risk % No Lymphopenia	Risk Difference (95% CI)	RR (95% CI)	*p*-Value	OS % Lymphopenia	OS % No Lymphopenia	HR (95% CI)	*p*-Value (Log-Rank)
Prior radiotherapy	65.1%	57.9%	7.1% (1.7–12.5)	1.12 (1.03–1.23)	0.0100	20.1%	26.0%	1.28 (1.11–1.48)	0.0006
Prior chemotherapy	62.7%	55.4%	7.3% (4.7–10.0)	1.13 (1.08–1.18)	<0.0001	23.6%	30.8%	1.24 (1.15–1.33)	<0.0001
Prior chemo-radiotherapy	63.3%	58.6%	4.7% (−1.4–10.9)	1.08 (0.98–1.20)	0.1281	21.4%	26.0%	1.19 (1.02–1.40)	0.0298

Subgroups defined by prior systemic therapy and/or radiation before immune checkpoint inhibitor initiation. Patients with death prior to the start of the 24-month analysis window were excluded from subgroup analyses (radiation subgroup: 10 vs. 10; chemotherapy subgroup: 37 vs. 36; chemo-radiation subgroup: 10 vs. 10 in lymphopenia vs. no-lymphopenia cohorts, respectively). OS = overall survival; HR = hazard ratio; RR = risk ratio; CI = confidence interval.

## Data Availability

All data (Tables and Figures) are presented in the body of the manuscript.

## Supplementary Materials

The following supporting information can be downloaded at: https://www.mdpi.com/article/10.3390/cancers18121940/s1, Figure S1: Propensity score distribution before and after matching in the lymphopenia and no-lymphopenia cohorts; Table S1: Codes used in cohort, exposure, comorbidity, laboratory definitions, and outcomes.
