# Supplementary material for: Baseline Lymphopenia Predicts Survival in ICI-Naïve Solid Tumor Patients Receiving Immune Checkpoint Inhibitors: A Propensity-Matched Real-World Pan-Cancer Analysis
Source: Cancers (Basel). 2026 Jun 14;18(12):1940. doi: 10.3390/cancers18121940 (PMC13297561; doi:10.3390/cancers18121940)
Supplement: Supplementary file 1 [file cancers-18-01940-s001.zip › cancers-4360497-supplementary.pdf]

Supplementary Table S1. Codes used in cohort, exposure, comorbidity, laboratory definitions, and outcomes.

Category	Description	Code system
**Demographics**		
Age	Age at index (≥18 years)	TriNetX (built-in)
Sex	Female, Male	TriNetX (built-in)
Race	White	LOINC 2106-3
	Black or African American	LOINC 2054-5
	Asian	LOINC 2028-9
	American Indian or Alaska Native	LOINC 1002-5
	Native Hawaiian or Other Pacific Islander	LOINC 2076-8
Ethnicity	Hispanic or Latino	LOINC 2135-2
	Not Hispanic or Latino	LOINC 2186-5
**Cohort Definition**		
PD-1 inhibitor	pembrolizumab	RxNorm 1547545
	atezolizumab	RxNorm 1792776
	nivolumab	RxNorm 1597876
Lymphocytes [#/volume] in Blood	TriNetX lab group	LG32863-9
**Tumor site**		
Malignant neoplasms of lip, oral cavity and pharynx	ICD-10-CM	C00–C14
Malignant neoplasms of digestive organs	ICD-10-CM	C15–C26
Malignant neoplasms of respiratory and intrathoracic organs	ICD-10-CM	C30–C39
Bronchial and Lung	ICD-10-CM	C34
Non-Small Cell Lung Cancer (NSCLC)	TNX Curated	TNX Curated
Malignant neoplasms of bone and articular cartilage	ICD-10-CM	C40–C41
Melanoma and other malignant neoplasms of skin	ICD-10-CM	C43–C44
Melanoma	ICD-10-CM	C43
Malignant neoplasms of mesothelial and soft tissue	ICD-10-CM	C45–C49
Malignant neoplasms of breast	ICD-10-CM	C50
Malignant neoplasms of female genital organs	ICD-10-CM	C51–C58
Malignant neoplasms of male genital organs	ICD-10-CM	C60–C63
Malignant neoplasms of urinary tract	ICD-10-CM	C64–C68
Malignant neoplasms of eye, brain and other parts of central nervous system	ICD-10-CM	C69–C72
Malignant neoplasms of thyroid and other endocrine glands	ICD-10-CM	C73–C75
Malignant neoplasms of ill-defined, other secondary and unspecified sites	ICD-10-CM	C76–C80
**Exclusion**		
Malignant neoplasms of lymphoid, hematopoietic and related tissue	ICD-10-CM	C81–C96
**Comorbidity**		
Diabetes mellitus	ICD-10-CM	E08–E13
Chronic kidney disease (CKD)	ICD-10-CM	N18

Alcoholic liver disease	ICD-10-CM	K70
HIV disease	ICD-10-CM	B20–B20
Inflammatory polyarthropathies	ICD-10-CM	M05–M14
Heart failure	ICD-10-CM	I50
Other chronic obstructive pulmonary disease	ICD-10-CM	J44
Hypertensive diseases	ICD-10-CM	I10–I1A
Diseases of arteries, arterioles and capillaries	ICD-10-CM	I70–I79
Cerebrovascular diseases	ICD-10-CM	I60–I69
Other degenerative diseases of the nervous system	ICD-10-CM	G30–G32
Personal history of irradiation	ICD-10-CM	Z92.3
**Medications**		
Antineoplastic agents	ATC	L01
Corticosteroids	ATC	H02AB
Immunosuppressants	ATC	L04A
Antibiotics, intestinal	ATC	A07AA
Proton pump inhibitors	ATC	A02BC
**Laboratory**		
Leukocytes [#/volume] in Blood	TriNetX lab	9015
Lactate dehydrogenase [enzymatic activity/volume] in Serum/Plasma	TriNetX lab	9052
Creatinine [mass/volume] in Serum/Plasma/Blood	TriNetX lab	9024
Neutrophils [#/volume] in Blood	TriNetX lab	9018
**Outcome**		
Deceased (mortality)	TriNetX demographics	Deceased flag
Healthcare utilization	HHT, outpatient, ED, observation, Inpatient, and ICU.	HCPCS G2176; CPT 1013675; SNOMED CT 394656005; SNOMED CT 32485007; HL7 V3 VisitType IMP; HL7 V3 VisitType OBSENC; SNOMED CT 305351004; LOINC 95420-6; CPT 1013729; CPT 1013711; LOINC 59258-4; SNOMED CT 4525004; CPT 99283; CPT 99284; CPT 99282; CPT 99281; CPT 99285; HL7 V3 VisitType EMER; HL7 V3 VisitType SS; HL7 V3 VisitType NONAC; HL7 V3 VisitType HH.
irAEs	Autoimmune diseases	ICD-10-CM K52.9; K51; K50; K52.89; A09; J84.89; J84.9; J84.112; J70.2; J70.4; K75.9; K75.3; K71.6; K71.9; K72.0; K76.89; E06.3; E06.9; E03.9; E05.90; E27.4; E27.49; E27.1; E23.0; E23.6; E10.9; E10.65; R73.9; N12; N10; N11.9; N14.0; N14.4; L27.0; L27.1; L50.9; L30.9; L51.9.
Gastrointestinal irAEs	ICD-10-CM	K52.9; K51; K50; K52.89; A09; K75.9; K75.3; K71.6; K71.9; K72.0; K76.89.
Pulmonary irAEs	ICD-10-CM	J84.89; J84.9; J84.112; J70.2; J70.4.
Endocrinology irAEs	ICD-10-CM	E06.3; E06.9; E03.9; E05.90; E27.4; E27.49; E27.1; E23.0; E23.6; E10.9; E10.65; R73.9.

Dermatology irAEs	ICD-10-CM	L27.0; L27.1; L50.9; L30.9; L51.9.
Infections	Pneumonia, sepsis, opportunistic infections	ICD-10-CM J12; J13; J14; J15; J16; J17; J18; A41; R65.20; R65.21; B37; B59; B00; B20; B44.

ATC, anatomical therapeutic chemical classification system; CKD, chronic kidney disease; CNS, central nervous system; COPD, chronic obstructive pulmonary disease; CT, computed terminology (SNOMED CT); ED, emergency department; GI, gastrointestinal; HCPCS, Healthcare Common Procedure Coding System; HIV, human immunodeficiency virus; HL7 V3, Health Level Seven version 3; ICD-10-CM, International Classification of Diseases, Tenth Revision, Clinical Modification; ICU, intensive care unit; ICI, immune checkpoint inhibitor; irAE, immune-related adverse event; LOINC, Logical Observation Identifiers Names and Codes; PD-1, programmed cell death protein-1; PD-L1, programmed death-ligand 1; RxNorm, normalized names for clinical drugs (U.S. National Library of Medicine); SD, standard deviation; TriNetX, global federated real-world data research network.

Supplementary Figure S1. Propensity score distribution before and after matching in the lymphopenia and no-lymphopenia cohorts.

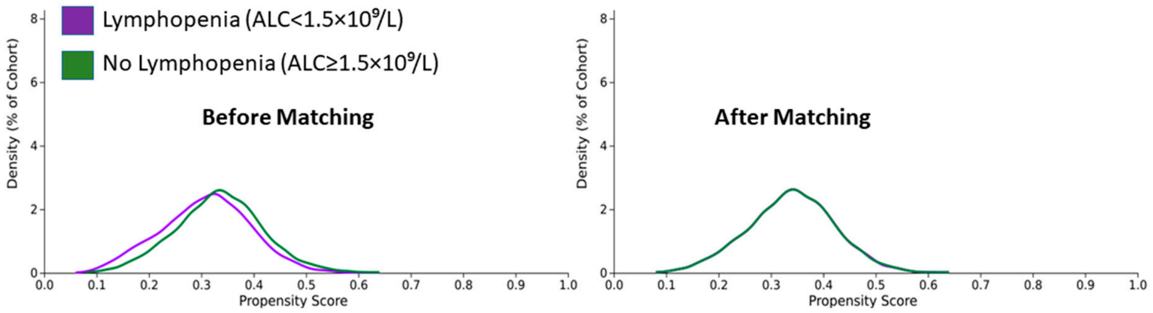

The figure shows the distribution of propensity scores in the lymphopenia and no-lymphopenia cohorts before and after 1:1 propensity score matching. Before matching, there was a clear separation between groups, consistent with baseline differences. After matching, substantial overlap in propensity score distributions was observed, indicating effective covariate balance and reduced confounding.
